# Antagonism of regulatory ISGs enhances the anti-melanoma efficacy of STING agonists

**DOI:** 10.3389/fimmu.2024.1334769

**Published:** 2024-01-18

**Authors:** Jessica N. Filderman, Jennifer L. Taylor, Jianmin Wang, Yali Zhang, Prashant Singh, Mark A. Ross, Simon C. Watkins, Ayah Nedal Al Bzour, Lilit Karapetyan, Pawel Kalinski, Walter J. Storkus

**Affiliations:** ^1^ Department of Immunology, University of Pittsburgh School of Medicine, Pittsburgh, PA, United States; ^2^ Department of Dermatology, University of Pittsburgh School of Medicine, Pittsburgh, PA, United States; ^3^ Department of Biostatistics and Bioinformatics, Roswell Park Comprehensive Cancer Center, Buffalo, NY, United States; ^4^ Genomics Shared Resource, Roswell Park Comprehensive Cancer Center, Buffalo, NY, United States; ^5^ Center for Biologic Imaging, University of Pittsburgh School of Medicine, Pittsburgh, PA, United States; ^6^ Department of Cell Biology, University of Pittsburgh School of Medicine, Pittsburgh, PA, United States; ^7^ Department of Medicine, Jordan University of Science and Technology, Irbid, Jordan; ^8^ Department of Cutaneous Oncology, H. Lee Moffitt Cancer Center and Research Institute, Tampa, FL, United States; ^9^ Department of Immunology, Roswell Park Comprehensive Cancer Center, Buffalo, NY, United States; ^10^ Department of Pathology, University of Pittsburgh School of Medicine, Pittsburgh, PA, United States; ^11^ Department of Bioengineering, University of Pittsburgh School of Medicine, Pittsburgh, PA, United States; ^12^ Cancer Immunology and Immunotherapy Program, UPMC Hillman Cancer Center, Pittsburgh, PA, United States

**Keywords:** ARG2, combination immunotherapy, COX2, immune checkpoint, ISG15, melanoma, NOS2, PTGS2

## Abstract

**Background:**

Stimulator of Interferon Genes (STING) is a dsDNA sensor that triggers type I inflammatory responses. Recent data from our group and others support the therapeutic efficacy of STING agonists applied intratumorally or systemically in a range of murine tumor models, with treatment benefits associated with tumor vascular normalization and improved immune cell recruitment and function within the tumor microenvironment (TME). However, such interventions are rarely curative and STING agonism coordinately upregulates expression of immunoregulatory interferon-stimulated genes (ISGs) including *Arg2, Cox2, Isg15, Nos2*, and *Pdl1* that may limit treatment benefits. We hypothesized that combined treatment of melanoma-bearing mice with STING agonist ADU-S100 together with antagonists of regulatory ISGs would result in improved control of tumor growth vs. treatment with ADU-S100 alone.

**Methods:**

Mice bearing either B16 (BRAF^WT^PTEN^WT^) or BPR20 (BRAF^V600E^PTEN^-/-^) melanomas were treated with STING agonist ADU-S100 plus various inhibitors of ARG2, COX2, NOS2, PD-L1, or ISG15. Tumor growth control and changes in the TME were evaluated for combination treatment vs ADU-S100 monotherapy by tumor area measurements and flow cytometry/transcriptional profiling, respectively.

**Results:**

In the B16 melanoma model, we noted improved antitumor efficacy only when ADU-S100 was combined with neutralizing/blocking antibodies against PD-L1 or ISG15, but not inhibitors of ARG2, COX2, or NOS2. Conversely, in the BPR20 melanoma model, improved tumor growth control vs. ADU-S100 monotherapy was only observed when combining ADU-S100 with ARG2i, COX2i, and NOS2i, but not anti-PD-L1 or anti-ISG15. Immune changes in the TME associated with improved treatment outcomes were subtle but included increases in proinflammatory innate immune cells and activated CD8^+^CD69^+^ T cells and varied between the two tumor models.

**Conclusions:**

These data suggest contextual differences in the relative contributions of individual regulatory ISGs that serve to operationally limit the anti-tumor efficacy of STING agonists which should be considered in future design of novel combination protocols for optimal treatment benefit.

## Introduction

Although melanoma is the least common form of skin cancer, it is the deadliest, with the number of cases steadily increasing over the past 30 years (1). The American Cancer Society has estimated that there were 97,610 new cases of melanoma in the US in 2023, resulting in 7,990 deaths (1). When melanoma is diagnosed early, it has proven comparatively easy to treat. However, once melanomas have metastasized, the 5-year survival rate of patients is only 15-20% (1, 2). Treatment with immune checkpoint inhibitors, such as anti-PD1, anti-PD-L1, anti-CTLA-4, and anti-LAG3, in the setting of late-stage melanoma has significantly improved the long-term survival of patients vs. previous standard-of-care therapies. However, 50-80% of patients fail to realize durable benefits from intervention with checkpoint blockade owing to a range of immune evasion mechanisms, including those that limit immune cell infiltration and the durability of anti-tumor T cells within the TME (3–7). As such, the need to develop more effective (combination) immunotherapies for cancer patients, particularly those with checkpoint refractory disease, remains a major unmet clinical priority.

One class of agents being developed for use in such treatment approaches is Stimulator of Interferon Genes (STING) agonists. STING is a cytosolic dsDNA sensor that triggers type I inflammatory responses (8). Upon binding to cGAMP, STING activates TBK1, which in turn phosphorylates IRF3 (8). Phosphorylated IRF3 homodimerizes and translocates into the nucleus where it transactivates IFNβ gene expression, along with other proinflammatory genes (8). Although STING is a prototypic component in the immune-mediated detection of viral infections, recent work has investigated the use of STING agonists as therapeutic drugs in the cancer setting. Within the TME, STING pathway activation leads to increases in Type I interferon signaling, the maturation of antigen presenting cells (APCs), and improved recruitment of T cells into tumors in support of slowed disease progression or tumor regression (9). Indeed, previous work from our group and others have shown that STING agonists are effective therapeutic agents in the setting of melanoma (10), colon cancer (11), prostate cancer (12), and pancreatic cancer (13), among others.

In murine melanoma models, we have previously shown that STING agonist ADU-S100 promotes vascular normalization (VN), as demonstrated by increased vascular perfusion, enhanced pericyte coverage of blood vessels, development of high endothelial venules (HEV) and lymphangiogenesis in treated tumors (10). These treatment-associated changes in the tumor vasculature facilitate improved immune cell entry and corollary formation of tertiary lymphoid structures (TLS) in the TME (10). Through TLS neogenesis and local expansion of unique anti-tumor T cell clonotypes not found in the periphery, ADU-S100 supports an immune-mediated delay in B16-F10 tumor growth and extended overall survival (10). However, such STING agonist-based therapies rarely provided durable systemic anti-tumor benefits (10). One potential mechanism underlying treatment resistance in this model involves therapy-induced upregulation of multiple interferon-stimulated genes (ISGs) within the TME that represent well-known immunoregulatory molecules, including ARG2, COX2/PTGS2, NOS2, and PD-L1 (10). These molecules restrict protective immune responses through diverse mechanisms which coordinately serve to limit anti-tumor immune effector cell function or fate within cancer lesions (14–19).

In the current report, we investigated the hypothesis that superior STING agonist-based immunotherapies can be achieved by antagonism/blockade of immunoregulatory ISGs. Indeed, in our preclinical tumor models we observed that the anti-tumor benefit of treating subcutaneously (s.c.) established B16 (BRAF^WT^PTEN^WT^) and BPR20 (BRAF^V600E^PTEN^-/-^) melanomas in C57BL/6 mice with locally delivered ADU-S100 was improved by cotreatment with either a combination of ARG2/COX2/NOS2 pharmacologic inhibitors or with neutralizing/blocking antibodies against PD-L1 or ISG15 (a molecule that can mediate either pro- or anti-tumor effects as an extracellular cytokine-like mediator based on microenvironmental context (20)). Remarkably, we found that different ISGs underlie resistance to ADU-S100 in different melanoma models, as B16 melanomas were most effectively treated with ADU-S100 + anti-PD-L1 or anti-ISG15 antibody, while BPR20 tumors were best treated with a combination of ADU-S100 + ARG2i/COX2i/NOS2i. TME profiling revealed that the preferential superiority of the combination treatment regimens in the B16 melanoma model was associated with improved levels of tumor infiltration by mature DC, M1 macrophages, and activated CD8^+^ T cells. In the BPR20 model, we observed higher infiltration of CD45^+^ immune cells and effector CD8^+^ T cells, as well as increased gene transcripts associated with inflammation and an anti-tumor immune response. These results suggest that the anti-tumor efficacy of STING agonist-based immunotherapy may be improved by combination treatment with regulatory ISG antagonists *in vivo*. Our findings also highlight divergence in the molecular mechanisms underlying resistance to STING agonists in genomically distinct melanomas suggesting consideration of more personalized approaches for optimal anti-tumor efficacy in prospective STING agonist-based clinical trials.

## Materials and methods

### Antibodies and pharmacologic inhibitors

The following antibodies were use in this study: blocking anti-mPD-L1 (10F.9G2, Rat IgG2b) antibody from BioXCell (Lebanon, NH) and blocking/neutralizing rabbit anti-h/mISG15 polyclonal antibody from G-Biosciences (St. Louis, MO). Species/Isotype control antibodies were purchased from Jackson ImmunoResearch Laboratories (West Grove, PA). CB-1158 (ARG2i) was purchased from ChemieTek (Indianapolis, IN). L-NMMA (NOS2i) was purchased from Millipore Sigma (St. Louis, MO). The COX2i Celecoxib was purchased from MedChem Express (Houston, TX).

### Tumor cell lines

The B16.F10 (CRL-6475) murine melanoma cell line was purchased from ATCC (Manassas, Virginia, USA) and passaged under sterile culture conditions. B16.F10 cells were grown at 37°C under 5% CO_2_ in RPMI-1640 supplemented with 10% heat-inactivated fetal bovine serum, 100 U/mL penicillin/streptomycin, and 10 mM L-glutamine (all components from GIBCO/ThermoFisher Scientific). BPR20 (BRAF^V600E^PTEN^-/-^) melanoma cells were derived from the BP melanoma cell line (21) (the kind gift of Dr. Jennifer Wargo, MD Anderson Cancer Center) and maintained under *in vitro* selection with 20 μM Dabrafenib (Selleck Chemicals) in DMEM supplemented with 10% heat-inactivated fetal bovine serum, 100 U/mL penicillin/streptomycin, and 10 mM L-glutamine (all components from GIBCO/ThermoFisher Scientific). Both cell lines were confirmed as mycoplasma-negative by RT-PCR as previously described (21).

### Animal models

Female C57Bl/6J mice (6-8 weeks old) were purchased from Jackson Laboratory (Bar Harbor, Maine, USA). Mice were injected subcutaneously (s.c.) in the right flank with either 10^5^ B16.F10 or 3.5 x 10^5^ BPR20 melanoma cells in 100 μL of PBS and allowed to establish solid tumors. After ~10 days (as indicated in text), palpable tumors were measured with calipers and mice were randomized into treatment groups with comparable mean tumor sizes. Mice were then injected intratumorally (i.t.) with sterile phosphate-buffered saline (PBS) or 5 μg of endotoxin-free ADU-S100 (Cat. No: HY-12885B, MedChemExpress) resuspended in 50 μl of sterile PBS. Repeat injections were administered 4 days later. Where indicated, mice were additionally dosed by oral gavage with 0.3 mg CB1158, 0.3 mg L-NMMA, and 1.2 mg celecoxib in a 20% Kolliphor solution for 5 days beginning on day 1 of treatment, followed by two days without dosing, and then an additional 5 days of dosing. For studies of combination therapy with PD-L1 antagonist, mice were injected i.p. with anti-PD-L1 (10 or 100 μg) on the initial day of treatment with ADU-S100 and then again 3, 7, and 10 days later. For ISG15 neutralization experiments, mice were injected i.t. on the initial day of treatment with 30 μg anti-ISG15, in combination with ADU-S100, with repeat dosing 4 days later. A third dose of anti-ISG15 antibody alone was administered 3 days later.

Tumor growth was measured every 2-3 days using a Vernier caliper. Tumor growth is reported as tumor area (in mm^2^ ± SD) based on the product of orthogonal measurements of the long and short axes of palpable tumors. All mice were monitored, treated, and euthanized (CO_2_ asphyxiation followed by cervical dislocation) under an Institutional Animal Care and Use Committee (IACUC)-approved protocol (# 21018759) per University of Pittsburgh’s Division of Laboratory Animal Resources (DLAR) recommended guidelines.

### Tumor harvest and processing

Tumors were resected on the day of euthanasia and digested using a cocktail of enzymes [RPMI containing 20 U/mL DNAse I (Sigma Chemicals, St. Louis, MO), 0.5 mg/mL Collagenase IA (Sigma), 0.5 mg/mL Collagenase II (Sigma), and 0.5 mg/mL Collagenase IV (Sigma)] for 30 min at 37°C on a shaker. Tumor digests were then passed through a 70 μm filter, with single cells washed twice using PBS prior to use in flow cytometry or sequencing.

### Flow cytometry

Cells were incubated at 4°C with fixable live/dead aqua dye (Life Technology) for 20 minutes and then washed with PBS + 2% FBS. Cells were then treated with FcR block (BD Biosciences) for 10 minutes at RT prior to a 20-minute incubation with primary antibodies at 4°C. Samples used for intracellular staining were fixed and permeabilized with the FoxP3 fix/perm kit (BD Biosciences) prior to incubation at RT for 30 minutes with antibodies targeting intracellular proteins. Flow analyses were performed using either BD LSR II or BD LSRFortessa flow cytometers housed within the Unified Flow Cytometry Core at the University of Pittsburgh. Flow cytometry data were acquired using BD FACSDiva software and analyzed using FlowJo V.10. The antibodies used in flow-based studies are listed in **Supplementary Table 1** with the gating strategies depicted in **Supplementary Figure 1**.

### Immunofluorescence microscopy (IFM)

Resected tumors were processed and stained per protocols established by the University of Pittsburgh’s Center for Biologic Imaging (www.cbi.pitt.edu) as previously described (10) using antibodies listed in **Supplementary Table 1**. Fluorescence images were acquired on an Olympus Provis, with image quantitation performed using Nikon Elements AR software and post-acquisition statistical analyses performed using GraphPad Prism software (La Jolla, CA).

### Gene array analysis

Resected tumors were enzymatically dissociated into single-cell suspensions and then frozen in RLT buffer at -80°C. RNA was isolated from the cell lysates using the RNEasy Micro Plus Kit (Qiagen) according to the manufacturer’s protocol. Gene expression analyses were performed within the Genomics Shared Resource at Roswell Park and the UPMC Hillman Cancer Center Cytometry facility using the nCounter mouse PanCancer Immune Profiling Panel (Nanostring) and the nCounter mouse Tumor Signaling 360 Panel (Nanostring), respectively, according to the manufacturer’s instructions. Data was analyzed by the Department of Biostatistics and Bioinformatics at the Roswell Park Comprehensive Cancer Center. Sample relationships were explored using PCA analysis and hierarchical clustering, with outliers excluded from the downstream analysis of the PanCancer Immune Profiling Panel. Differential gene expression analysis was performed using DESeq2 (22) using the normalized data, with an FDR cutoff of 0.05. Treatment effect, mouse model (B16 and BPR20) effect, and their interactions were identified using a factorial design model. Gene expression results are available on the GEO database (GSE249296).

### Statistical analyses

Comparisons between two groups were performed using two-tailed Student’s t-tests, while comparisons between multiple groups were performed using one-way or two-way analysis of variance (ANOVA) with Tukey’s *post hoc* analysis as indicated in text. Inter-group differences with P values < 0.05 were considered significant. Prism V.10 (GraphPad) was used to generate graphs and perform statistical tests.

## Results

### Differentially expressed genes (DEG) associated with the anti-tumor efficacy of i.t. STING agonist ADU-S100 immunotherapy in B16 and BPR20 melanoma models include regulatory ISGs

**Figure 1:**
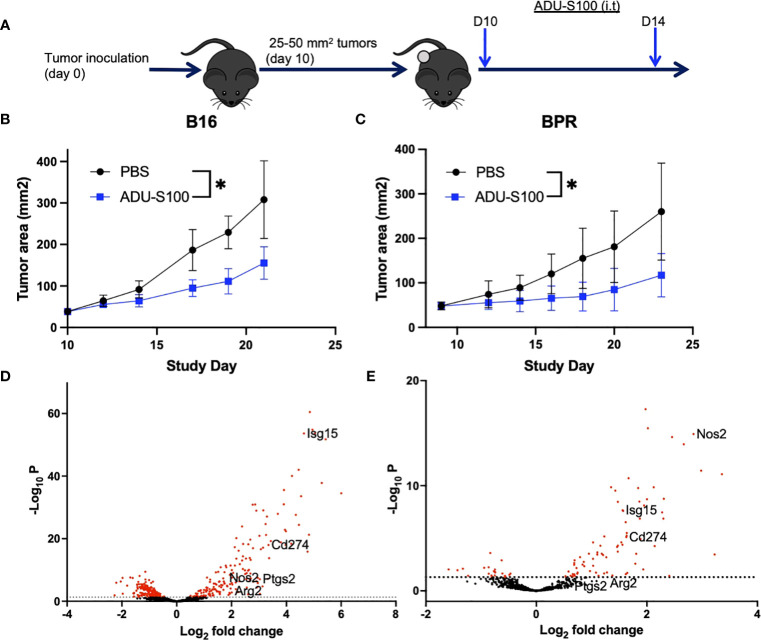
Treatment with ADU-S100 leads to improved tumor growth control and changes in tumor-associated gene profiles. **(A)** Mice bearing established s.c. B16-F10 **(B)** or BPR20 **(C)** melanomas were treated i.t. with ADU-S100 vs. PBS (n = 5 mice/group) as outlined in Materials and Methods and tumor growth was monitored over time (*p<0.05 for both models, paired t-test). Six hours after the second dose of ADU-S100, mRNA was isolated from the tumors and analyzed using the nCounter mouse PanCancer Immune Profiling Panel. Differentially expressed genes (DEG) from untreated vs ADU-S100-treated B16 **(D)** and BPR20 **(E)** are indicated in Volcano plots. The ISGs *Arg2, Isg15, Nos2*, and *Pdl1* were identified as DEGs coordinately upregulated in both tumor models after treatment with ADU-S100, while *Cox2/Ptgs2* was only significantly upregulated in the B16 model. Rank ordered DEG expression for each melanoma model is listed in **Supplementary Table 2**.

Intratumoral delivery of ADU-S100 (**Figure 1A**) slowed the growth of s.c. B16 (BRAF^WT^PTEN^WT^) and BPR20 (BRAF^V600E^PTEN^-/-^) melanomas in syngeneic C57BL/6 mice (**Figures 1B**, **C**). Transcriptional profiling of the TME of ADU-S100 treated vs. untreated tumors identified therapy-associated DEGs in each model which included ISGs (**Figures 1D**, **E**, **Supplementary Table 2**) known to mediate pro-tumor effects. Notably, the rank order of ADU-S100-induced immunoregulatory DEGs was different when comparing treated B16 (*Isg15* > *Pdl1/Cd274* > *Cox2/Ptgs2* > *Arg2* > *Nos2*) vs. BPR20 (*Nos2* > *Pdl1/Cd274* > *Isg15* > *Arg2*) melanomas (**Supplementary Table 2**), suggesting intrinsic differences in STING pathway signaling between these unrelated *in vivo* tumor models. While therapeutic STING agonism coordinately enhanced expression of the *Arg2, Isg15, Nos2*, and *Pdl1* (*Cd274*) immuno-suppressive/regulatory ISGs in both melanoma models, *Cox2/Ptgs2* expression was selectively upregulated by ADU-S100 treatment only in the B16 model. Based on these findings, we hypothesized that optimal treatment benefits associated with the administration of STING agonist in our tumor models might be restrained by compensatory increases in expression of one or more of these (immuno)regulatory ISGs.

### Targeted neutralization/antagonism of regulatory ISGs in the TME enhances the anti-melanoma efficacy of ADU-S100 *in vivo*

**Figure 2:**
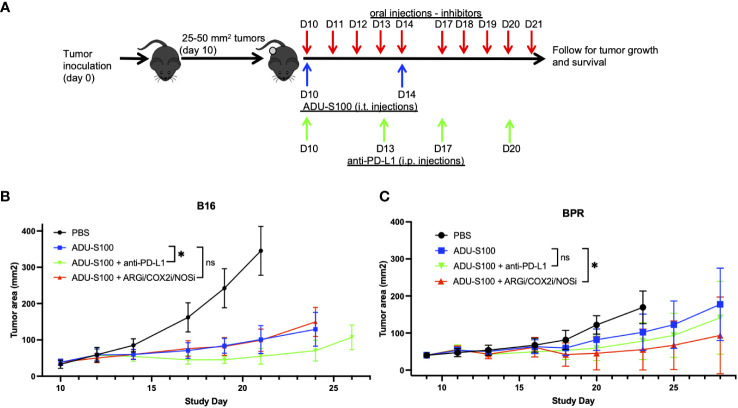
Combination treatment with ADU-S100 + regulatory ISG antagonists improves therapeutic anti-tumor efficacy versus ADU-S100 monotherapy, but in a melanoma model-dependent manner. **(A)** Mice with established B16 or BPR20 melanomas were treated with ADU-S100 +/- anti-PD-L1 antibody or a combination of pharmacologic inhibitors (i.e., ARG2i, COX2i, NOS2i). B16 **(B)** and BPR20 **(C)** tumor size was then monitored every 2-3 days. (n = 10, ns = not significant, *p<0.05, Two-Way ANOVA).

To determine whether ADU-S100-based immunotherapy could be improved by cotreatment with antagonists of regulatory ISGs, mice bearing established s.c. B16 or BPR20 melanomas were left untreated or they were treated with i.t. ADU-S100 alone or ADU-S100 combined with i.) ARG2i + COX2i/PTGS2i + NOS2i administered via oral gavage, or ii.) anti-PD-L1 blocking mAb injected i.p. (**Figure 2A**). Since individual applications of ARG2i, COX2i, and NOS2i failed to significantly impact B16 or BPR20 melanoma growth as monotherapies or when combined with ADU-S100 (data not shown), we instead provided these agents as a 3-agent antagonist cocktail. While combination ADU-S100 + ARG2i/COX2i/NOS2i immunotherapy failed to significantly enhance anti-tumor benefits vs. ADU-S100 alone in the B16 model (**Figure 2B**), this regimen was significantly superior to ADU-S100 alone in treating established BPR20 melanomas (**Figure 2C**). Despite the consensus that B16 melanomas are refractory to anti-PD-L1 monotherapy (10, 23), addition of anti-PD-L1 to the ADU-S100 treatment regimen yielded improved anti-tumor efficacy in this model (**Figure 2B**). Remarkably, this was not observed in the BPR20 melanoma model (**Figure 2C**), where treatment with ADU-S100 + anti-PD-L1 failed to reduce tumor growth vs. treatment with ADU-S100 alone. No loss of animal body weight, alteration in animal behavior, or other signs of adverse events were observed in any of the treatment groups vs. control (data not shown). These data suggest differences in the operational dominance of individual regulatory ISG-associated mechanisms across our two melanoma models as they relate to disease outcomes after treatment with ADU-S100.

### Antagonism of regulatory ISGs in combination treatment protocols results in altered changes in immune profiling within the TME vs. treatment with ADU-S100 alone

**Figure 3:**
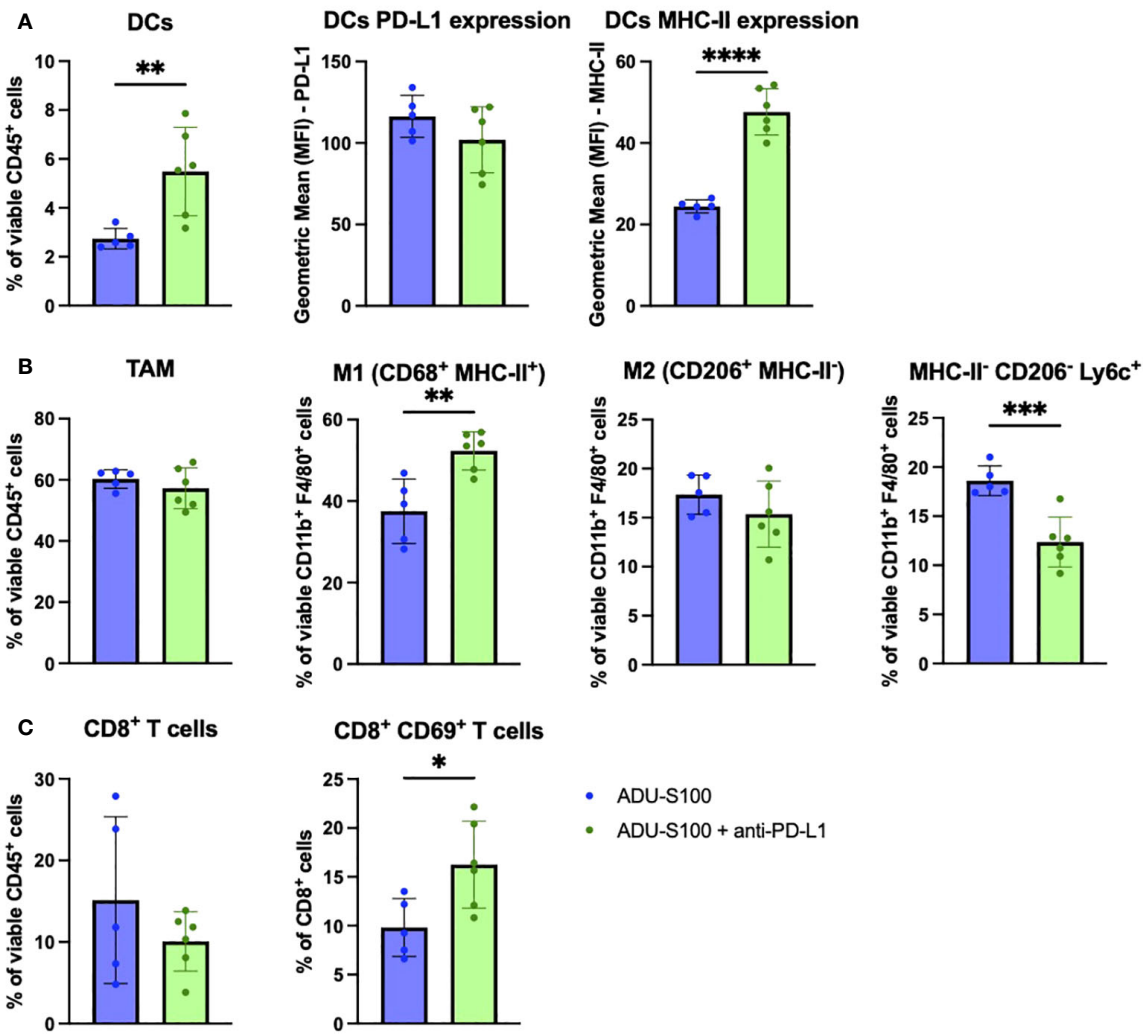
Treatment of established B16 melanomas with ADU-S100 + anti-PD-L1 results in proinflammatory changes in immune cell status in TME vs. treatment with ADU-S100 alone. Mice with established s.c. B16 melanomas were treated with ADU-S100 +/- anti-PD-L1 as outlined in **Figure 2A**. On day 7 after the initiation of treatment, tumors were harvested, dissociated into single cell suspensions, and stained for flow cytometry analysis as outlined in Materials and Methods, with a focus on **(A)** DCs, **(B)** TAM and M1/M2 macrophages, and **(C)** CD8^+^ TIL (n = 6, *p<0.05, **p<0.01, ****p<0.0001, unpaired two-tailed Student’s t-test).

Given the observed improvement in tumor growth control by supplementing ADU-S100-based immunotherapy with anti-PD-L1 antibody in the B16 melanoma model, and by combining ARG2i + COX2i + NOS2i with ADU-S100 in the BPR20 melanoma model, we next evaluated correlative changes in the therapeutic TME in both systems. We assessed single cells isolated from tumors 7 days after initiating therapy by flow cytometry. Levels of tumor-associated CD45^+^ immune cells did not significantly change in frequency in B16 melanomas receiving combined treatments vs. ADU-S100 alone (**Supplementary Figure 2**). However, we did observe a significant increase in the frequency of CD11c^+^ DCs in B16 tumors treated with ADU-S100 + anti-PD-L1 compared to ADU-S100 alone, with these antigen presenting cells appearing more mature based on their expression of significantly higher levels of MHC-II molecules (**Figure 3A**). The total percentage of CD11b^+^F4/80^+^ tumor-associated macrophages (TAMs) did not change between the treatment groups, but the percentage of M1 (CD68^+^MHC-II^+^) macrophages was significantly increased in the ADU-S100 + anti-PD-L1 therapy cohort (**Figure 3B**). No significant differences were observed in the frequency of M2 (CD206^+^MHC-II^-^) macrophages in the TME when comparing ADU-S100 monotherapy vs. combination treatment; however, there was a significant decrease in the frequency of MHC-II^-^CD206^-^Ly6c^+^ monocytes found in combination treated tumors (**Figure 3B**). These changes in M1/M2 infiltration of the TME were also observed in IFM analyses of tumor sections (**Supplementary Figure 3**). There was a significant increase in the frequency of M-MDSC (CD11b^+^F4/80^-^Ly6c^+^Ly6g^-^) in ADU-S100 + anti-PD-L1 treated B16 tumors, with these cells expressing reduced levels of PD-L1 when compared to their counterparts in B16 tumors treated with ADU-S100 alone (**Supplementary Figures 4A, B**). Interestingly, while PMN-MDSC (CD11b^+^Ly6g^+^) frequencies were not altered between the treatment cohorts, PD-L1 expression on PMN-MDSCs was reduced in B16 melanomas treated with ADU-S100 + anti-PD-L1 vs. ADU-S100 alone (**Supplementary Figure 4B**). These results suggest that ADU-S100 + anti-PD-L1 intervention promotes a more pro-inflammatory, less regulatory myeloid compartment within the B16 TME in association with slowed tumor growth. Within the TIL compartment, no significant changes were observed in the frequency of CD8^+^ T cells, CD4^+^ T cells, or Tregs (CD4^+^FoxP3^+^) within the TME (**Figure 3C**, **Supplementary Figure 4**). However, the CD8^+^ TIL in ADU-S100 + anti-PD-L1 vs. ADU-S100 only treated B16 melanomas expressed significantly higher levels of early activation marker CD69, consistent with their enhanced functional status, but they exhibited no significant changes in Granzyme B or PD1 expression (**Figure 3C**, **Supplementary Figure 4D**). Additionally, B16 tumor cells expressed significantly less PD-L1 and significantly more MHC-II when treated with ADU-S100 + anti-PD-L1 compared to ADU-S100 alone (**Supplementary Figure 4E**), indicating that the combination therapy induced a more pro-inflammatory, less-immune evasive phenotype in melanoma cells as well.

**Figure 4:**
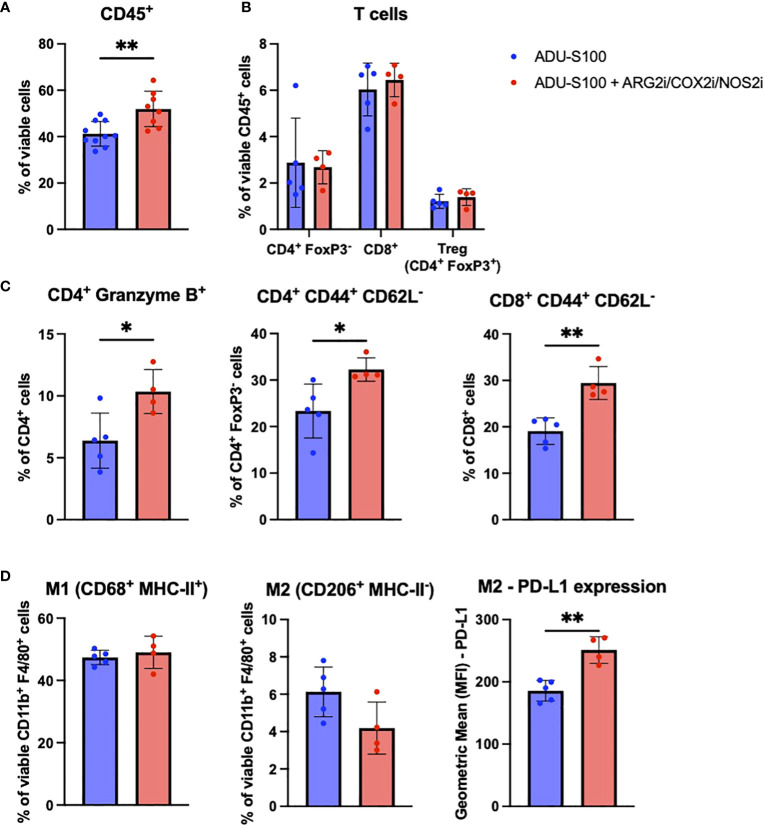
The addition of ARG2i/COX2i/NOS2i to ADU-S100-based immunotherapy leads to increased immune cell infiltration into BPR20 melanomas. Mice with established BPR20 tumors were treated with ADU-S100 +/- ARG2i/COX2i/NOS2i as described in **Figure 2A**. Tumors were harvested on day 7 post-treatment initiation, dissociated into single cell suspensions, and stain for flow cytometry. Cells that were evaluated include **(A)** CD45^+^ immune cells, **(B)** and **(C)** T cells, and **(D)** M1/M2 macrophages (n = 4-5, *p<0.05, **p<0.01, unpaired two-tailed Student’s t-test, 1 outlier was removed from the ADU-S100 only group and 2 outliers were removed from the ADU-S100 + ARG2i/COX2i/NOS2i group based on disparate PCA clustering analysis).

In the BPR20 model, we observed a significant increase in CD45^+^ immune cell infiltration into the TME after treatment with ADU-S100 + ARG2i/COX2i/NOS2i vs. ADU-S100 alone (**Figure 4A**). No significant changes were seen in the frequency of CD4^+^ T cells, CD8^+^ T cells, Tregs (**Figure 4B**) or other infiltrating immune cells in the TME (data not shown). However, we found that the CD4^+^ T cells present in the ADU-S100 + ARG2i/COX2i/NOS2i treated tumors expressed significantly higher levels of Granzyme B than their counterparts in single-agent ADU-S100 treated tumors (**Figure 4C**). Additionally, both CD4^+^ and CD8^+^ T cells had significantly higher frequencies of CD44^+^CD62L^-^ effector cells after treatment with ADU-S100 + ARG2i/COX2i/NOS2i (**Figure 4C**). In the myeloid compartment, we observed no changes in the M1 macrophage frequency and a trend toward a decrease in M2 macrophages in tumors treated with ADU-S100 + ARG2i/COX2i/NOS2i compared to treatment with ADU-S100 alone, with the M2 macrophages expressing higher levels of PD-L1 (**Figure 4D**). To further investigate changes occurring in tumor cells and immune cells after treatment in the BPR20 model, we implemented the Nanostring Tumor Signaling 360 panel analyses. We observed 88 genes significantly up-regulated and 43 genes significantly down-regulated in ADU-S100 + ARG2i/COX2i/NOS2i combination therapy vs. ADU-S100 monotherapy (**Supplementary Figure 5A**, **Supplementary Table 3**). Per Nanostring gene set classifications, many of the up-regulated DEGs play roles in inflammation and immune-mediated killing of tumors, while the majority of the down-regulated DEGs are involved in tumor invasion/metastasis, evasion of cellular growth suppression, genome instability and mutation, and sustained proliferative signaling. Performance of a pathway analysis on these data revealed coordinate up-regulation of anti-tumor immune-related pathways, including those involving immunoregulatory interactions between lymphoid and non-lymphoid cells, pro-inflammatory Th1 pathways, costimulation of effector cells mediated by CD28 family members, and the macrophage classical activation signaling pathway, among others (**Supplementary Figure 5B**).

### ADU-S100-based immunotherapy of B16 but not BPR20 melanomas is improved by i.t. delivery of blocking/neutralizing anti-ISG15 antibody

**Figure 5:**
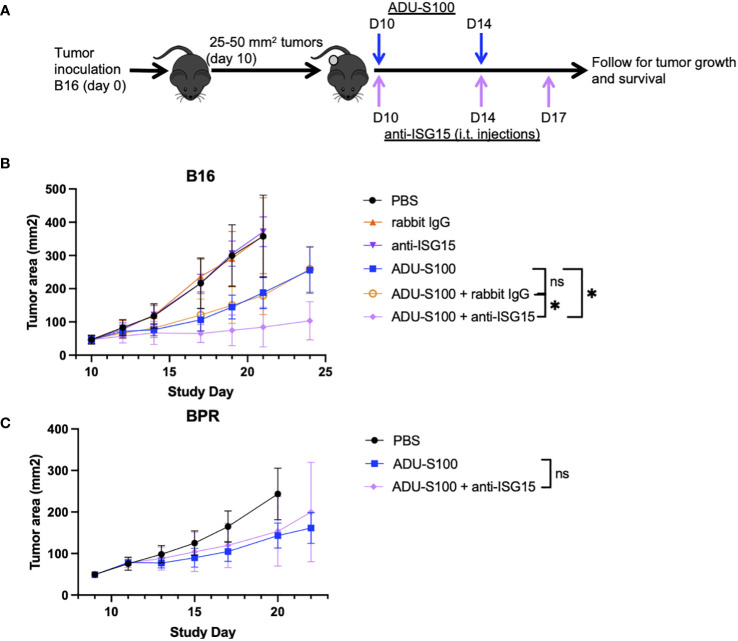
ADU-S100-based immunotherapy of B16 but not BPR20 melanomas is improved by i.t. delivery of blocking/neutralizing anti-ISG15 antibody. **(A)** Mice with established s.c. B16 or BPR20 melanomas were treated i.t. with ADU-S100 +/- neutralizing anti-ISG15 antibody as outlined in Materials and Methods. Tumor growth was then monitored in the **(B)** B16 and **(C)** BPR20 models over time (n = 5, *p<0.05, Two-Way ANOVA).

Since ISG15 has been reported to mediate both pro-inflammatory and immunoregulatory activities as an extracellular, cytokine-like molecule (20, 24–28), we further investigated whether the therapeutic efficacy of ADU-S100 could be improved by coadministration of blocking/neutralizing anti-ISG15 antibody in the B16 and BPR20 melanoma models (**Figure 5A**). As shown in **Figure 5B**, we observed that cotreatment with ADU-S100 + anti-ISG15 antibody resulted in improved control of B16 tumor growth vs. treatment with ADU-S100 monotherapy. In contrast, the growth of established BPR20 melanomas treated with ADU-S100 + anti-ISG15 antibody appeared indistinguishable from treatment with ADU-S100 alone (**Figure 5C**). These findings suggest intrinsic differences between the B16 and BPR20 melanomas regarding potential mechanisms underlying the effectiveness of STING agonist-based interventions, which may reflect variance in the operational regulatory action of ISG15 across these two unrelated tumor models.

**Figure 6:**
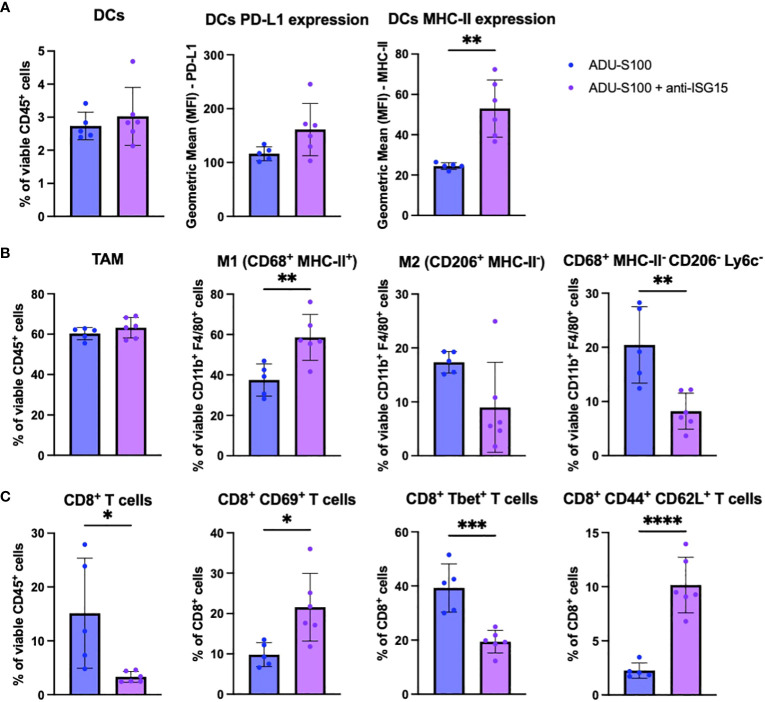
Immune correlates in the B16 TME associated with superior outcome after treatment with ADU-S100 + anti-ISG15 vs. ADU-S100 monotherapy. Mice with established s.c. B16 melanomas were treated as outlined in **Figure 4A**. On day 7 after the initiation of treatment, tumors were harvested, dissociated into single cell suspensions, and stained for flow cytometry analysis as outlined in Materials and Methods, focusing on **(A)** DCs, **(B)** TAM and M1/M2 macrophages, and **(C)** CD8^+^ TIL. (n = 6, *p<0.05, **p<0.01, ***p<0.001, unpaired two-tailed Student’s t test).

To investigate immune mechanisms underlying superior B16 growth inhibition after treatment with ADU-S100 + anti-ISG15 vs. ADU-S100 monotherapy, we profiled the phenotypes of cells found in the TME by flow cytometry. Akin to our observations for B16 tumors treated with ADU-S100 + anti-PD-L1 vs. ADU-S100 alone, we observed increased expression of MHC-II on CD11c^+^ DCs (**Figure 6A**), increased frequency of M1 macrophages (with no change in total macrophages or M2 macrophages; **Figure 6B**), and enhanced frequencies of M-MDSCs in B16 tumors treated with ADU-S100 + anti-ISG15 vs. ADU-S100 alone (**Supplementary Figure 6A**). These changes in the myeloid compartment were suggestive of skewing toward a more pro-inflammatory innate immune TME. In the TIL compartment, B16 melanomas treated with ADU-S100 + anti-ISG15 (vs. ADU-S100 alone) displayed a significant decrease in total CD8^+^ TIL content, but these T cells were enriched in the CD8^+^CD69^+^ activated phenotype (**Figure 6C**). Animals treated with ADU-S100 + anti-ISG15 had a significant increase in the frequency of tumor infiltrating CD62L^+^CD44^+^ central memory CD8^+^ and CD4^+^ T cells compared to those treated with ADU-S100 alone, as well as an increase in CD44^+^CD62L^-^ effector CD4^+^ TIL (**Figure 6C**, **Supplementary Figure 6C**). Furthermore, CD45^neg^ tumor/stromal cells in B16 tumors treated with ADU-S100 + anti-ISG15 (vs. ADU-S100 alone) expressed higher levels of MHC II, with a trend for increased expression of PD-L1 (p = 0.1042; **Supplementary Figure 6F**).

## Discussion

A major finding in the current report reflects the regulatory action of several ISGs (ARG2, ISG15, NOS2, PD-L1, PTGS2/COX2) that serve to limit the anti-tumor activity of STING agonists *in vivo*, with targeted ISG antagonists improving treatment outcomes when applied in combination with STING agonist ADU-S100. Remarkably, the dominance of individual regulatory ISGs in restricting ADU-S100 therapy benefits varied between the B16 and BPR20 melanoma models, with anti-PD-L1 and anti-ISG15 (but not ARG2i/COX2i/NOS2i) preferentially improving the therapeutic efficacy of STING agonist in the B16 (BRAF^WT^PTEN^WT^) melanoma model. Conversely, i.t. administration of ADU-S100 together with systemic oral delivery of ARG2i/COX2i/NOS2i (but not i.p. anti-PD-L1 or i.t. anti-ISG15) provided superior anti-tumor efficacy vs. ADU-S100 alone in the BPR20 (BRAF^V600E^PTEN^-/-^) melanoma model. While our results regarding therapeutic anti-tumor synergy between STING agonists and individual ARGi, COX2i, or ICI treatments are consistent with some previous reports (11, 29–34), they reinforce a critical need to consider intrinsic heterogeneity in tumors and their associated TMEs as important variables in estimating the likely efficacy of specific drug combinations in STING-based immunotherapies. Baseline expression levels of ISGs and genes associated with the cGAS-STING signaling pathway were investigated in both melanoma models, but these did not correlate with differential outcomes to combination therapies (**Supplementary Figure 7**).

Our observation that (ADU-S100 induced) ISG15 mediates pro-tumor effects in the TME that may be antagonized for therapeutic gain is novel. ISG15 is a major downstream product of STING activation, existing as both an intracellular ubiquitin-like protein (Ubl) and an extracellular cytokine/alarmin-like molecule (24, 25, 35). Notably, ISG15 has been reported to mediate both pro-inflammatory (early) and immunosuppressive (late) effects in a context-dependent manner. Forced expression models suggest that free extracellular ISG15 may initially limit tumor progression in an NK cell-dependent manner (36), with ISG15 also reported to serve as an effective vaccine adjuvant for CD8^+^ T cell crosspriming (37). However, extracellular ISG15 has also been posited to facilitate cytokine release syndrome (“cytokine storms”) which may negatively impact protective pro-inflammatory immune responses in association with immune related adverse events (38). A recent report by Chen et al. (24) further suggests that tumor released extracellular ISG15 enforces pro-tumorigenic M2 TAM via an LFA1-SFK-CCL18-dependent signaling pathway. Furthermore, melanoma shed ISG15 has been reported to promote E-cadherin expression on DCs which may negatively impact the migratory behavior of these potent APCs (26) important for T cell crosspriming in tumor-draining lymph nodes. Free ISG15 also appears to promote development of tolerogenic APCs (39) which support Treg generation (40) and may protect Treg from IFN-induced fragility (27), potentially sustaining Treg suppressor activity in the TME. Despite these intriguing potential mechanisms of immunoregulatory action, we failed to observe major changes in M2 TAM or Treg presence the TME of B16 tumors treated with ADU-S100 +/- blocking/neutralizing anti-ISG15 antibody, suggesting that ISG15 may mediate additional (as yet) undefined pro-tumor mechanisms in the B16 (but not BPR20) melanoma model.

Of potential clinical importance, elevated expression of ISG15 in the TME has been linked to higher histological grade, larger tumor size, a “stemmy” cancer phenotype, low CD8^+^ TIL content, expression of PD-L1/IDO-1/LAG-3, and poor clinical prognosis (41–46). Transcriptional profiling of public melanoma data sets revealed that *ISG15* is most highly expressed by macrophages in the TME (**Supplementary Figure 8A**), that *ISG15* expression is positively associated with expression of checkpoint molecules PD-1, PD-L1, and CTLA4 (**Supplementary Figure 8B**), and that high tumor expression of *ISG15* is associated with reduced objective response rate (ORR) with a trend for reduced overall survival (OS) (**Supplementary Figure 8C**). While these transcriptional profiling data cannot dissect the impact of intracellular (ISGylation-associated) vs. extracellular cytokine-like attributes on ISG15-related outcomes in melanoma patients, they are consistent with an immunoregulatory role for high expression levels of ISG15 in the TME. Our translational data suggests that extracellular ISG15, presumably acting as a cytokine-like molecule in the TME, mediates regulatory activity restraining optimal therapeutic benefit associated with therapeutic delivery of STING agonist ADU-S100, but only in some cases (i.e., in the B16 but not BPR20 melanoma model).

Phenotypic and transcriptional profiling of the therapeutic TME in B16 and BPR20 bearing mice treated with ADU-S100 + ISG antagonists revealed only modest changes in immune cell content in association with improved control of tumor growth when compared to ADU-S100 monotherapy. The most notable differences linked to combined treatment benefits included an increase in pro-inflammatory myeloid cell subsets, as well as more activated CD8^+^ T cells in B16 tumors treated with ADU-S100 plus either anti-PD-L1 or anti-ISG15 antibodies. B16 tumors treated with these combination regimens exhibited increased frequencies of CD68^+^MHC-II^+^ M1 macrophages and mature CD11c^+^MHC-II^+^DC when compared to tumors treated only with ADU-S100, suggesting that addition of anti-PD-L1 or anti-ISG15 antibodies to STING agonist-based protocols promotes a qualitatively superior pro-inflammatory/anti-tumor TME from an innate immune cell perspective. In ADU-S100 + anti-PD-L1 treated tumors, the frequency of MHC-II^-^CD206^-^Ly6c^+^ monocytes was significantly decreased (47, 48). As these monocytes are capable of differentiating into MHC-II^hi^ pro-inflammatory and MHC-II^lo^ TAMS (49), this finding leads us to believe that treatment with ADU-S100 + anti-PD-L1 may preferentially promote the differentiation of Ly6c^+^ monocytes into CD68^+^MHC-II^+^ M1 TAMs. It is important to note that our M1/M2 phenotyping of TAMs is based only on cell surface receptors and does not include other canonical markers, such as iNOS, Arg1, and VEGF. Further analysis of these cells is warranted to confirm their functional status. Although we observed an increase in inflammatory, anti-tumor myeloid cells, we also noted an increase in M-MDSCs in the B16 model after combined treatment with ADU-S100 and either anti-PD-L1 or anti-ISG15, indicating that regulatory/tolerogenic mechanisms may remain in play. M-MDSCs are known to be involved in resistance to anti-PD-L1 therapy (50, 51) and STING agonism has been previously shown to induce M-MDSC recruitment/activation in the TME (52, 53). Since we did not profile cytokines and chemokines associated with MDSC recruitment in our TME analysis, such analyses will represent a focus in future studies to more comprehensively understand compensatory regulatory mechanisms stimulated by STING agonism.

On the other hand, changes in TIL associated with superior anti-tumor efficacy for the combination STING-based immunotherapies in the B16 model were subtle and primarily linked to elevated frequencies of activated CD8^+^CD69^+^ T cells, with CD45^neg^ tumor/stromal cells coordinately expressing higher levels of MHC-II expression. In ADU-S100 + anti-ISG15 treated tumors, we observed an increase in the frequency of central memory CD4^+^ and CD8^+^ T cells in addition to an increase in effector CD4^+^ T cell content vs. ADU-S100 treatment alone, suggestive of enhanced anti-tumor T cell activity *in situ*. In BPR20 tumors treated with ARG2i/COX2i/NOS2i, an increase in CD45^+^ immune cell infiltration into the tumor was observed, corresponding with increases in gene transcripts associated with immune cell-mediated tumor growth control and inflammation (e.g., *Ccl21a, Fasl, Cd8b1, Cd40, Cd4, Cxcl11*). Additionally, genes typically involved with tumor growth and persistence were downregulated with the addition of ARG2i/COX2i/NOS2i to STING agonism (e.g., *Ar, Tgfb3, Tead2, Pik3r2, Jun*). While we did not observe the same skewing of macrophages toward M1 TAMs or more mature DCs in our BPR model, we did note an increase in cytotoxic Granzyme B^+^ CD4^+^ T cells known to mediate direct killing of MHC II+ tumor cells (54), as well as an increase in central memory CD4^+^ and CD8^+^ TIL.

Since our models inherently vary in tumor intrinsic BRAF^WT^ vs. BRAF^V600E^ expression as well as PTEN (WT vs. null) expression, it could be posited that these specific gene alterations influence differential response to regulatory ISG antagonists. Where such associations have been investigated in the clinical setting, melanoma BRAF mutation status has not been found to be predictive of patient response to anti-PD1/PD-L1 treatment (55–57), while PTEN loss in melanoma may predict inferior patient response to treatment with PD1/PD-L1 antagonists (57, 58). This latter finding is consistent with our observation for the ineffectiveness of anti-PD-L1 antibody to enhance the anti-tumor efficacy of ADU-S100 in the BPR20 (PTEN^-/-^) model. While no similar correlates have yet been reported for melanoma patient response to targeted inhibitors of regulatory ISGs based on tumor *BRAF/PTEN* gene expression stratification, at least for ISG15, our screening of a publicly accessible data set failed to reveal any correlation between BRAF mutation or PTEN presence/loss with *ISG15* high vs. low expression status in a melanoma cohort (**Supplementary Figure 8D**). Extended analyses of additional murine melanoma models would be required to generalize the regulatory roles of individual ISGs in response to STING agonist treatment based on tumor genotype/phenotype status.

Although early-generation STING agonists (including ADU-100) have shown great efficacy in pre-clinical studies, they require delivery into accessible tumor lesions and have demonstrated modest clinical efficacy (59). Given these known limitations, we plan to prospectively evaluate whether next-gen STING agonists that can be delivered systemically and which hold greater clinical promise (59) are susceptible to similar regulatory ISG tempering in their therapeutic efficacy. In these studies, we would expect heterogeneity in the operational dominance of individual ISGs across tumor models.

It is also worth noting that the B16 melanoma model was originally derived from a male mouse while the BPR20 model derives from a female mouse (21, 60). Androgens are known to play a role in modulating the immune system so it is possible that sexual dimorphism is also playing a role in the response to therapy between our two model systems (61, 62). Androgen receptor expression is the most significantly downregulated gene in the BPR20 model after treatment with ADU-S100 + ARG2i/COX2i/NOS2i (**Supplementary Table 3**), suggesting future interrogation of the role(s) of androgens/androgen receptors in limiting STING agonist-based treatment outcomes.

In conclusion, our findings support a paradigm in which regulatory ISGs limit the optimal anti-tumor effectiveness of immunotherapies implementing STING agonists such as ADU-S100, with the operationally relevant regulatory ISGs varying in a melanoma model-dependent manner which could have implications for heterogeneous responses in melanoma patients to STING agonists in the clinic. They further support translation of combination STING-based immunotherapies incorporating antagonists of one or more regulatory ISGs as an approach to provide improved treatment benefits to patients with melanoma.

## Data availability statement

The datasets presented in this study can be found in online repositories. The names of the repository/repositories and accession number(s) can be found below: GEO database (GSE249296).

## Ethics statement

The animal study was approved by University of Pittsburgh IACUC. The study was conducted in accordance with the local legislation and institutional requirements.

## Author contributions

JF: Conceptualization, Writing – original draft, Writing – review & editing, Data curation, Formal Analysis, Investigation, Methodology. JT: Data curation, Formal Analysis, Investigation, Writing – review & editing. JW: Formal Analysis, Writing – review & editing, Methodology. YZ: Formal Analysis, Methodology, Writing – review & editing. PS: Formal Analysis, Writing – review & editing, Conceptualization. MR: Writing – review & editing, Data curation, Investigation. SW: Writing – review & editing, Conceptualization, Formal Analysis. ANAB: Formal Analysis, Writing – review & editing. LK: Formal Analysis, Writing – review & editing. PK: Writing – review & editing, Conceptualization, Funding acquisition. WS: Conceptualization, Funding acquisition, Writing – review & editing, Project administration, Resources, Supervision, Writing – original draft.
