# Supplementary material for: Antagonism of regulatory ISGs enhances the anti-melanoma efficacy of STING agonists
Source: Front Immunol. 2024 Jan 18;15:1334769. doi: 10.3389/fimmu.2024.1334769 (PMC10835797; doi:10.3389/fimmu.2024.1334769)
Supplement: Supplementary file 1 [file Presentation_1.pdf]

Fig. S1, Filderman et al.

A

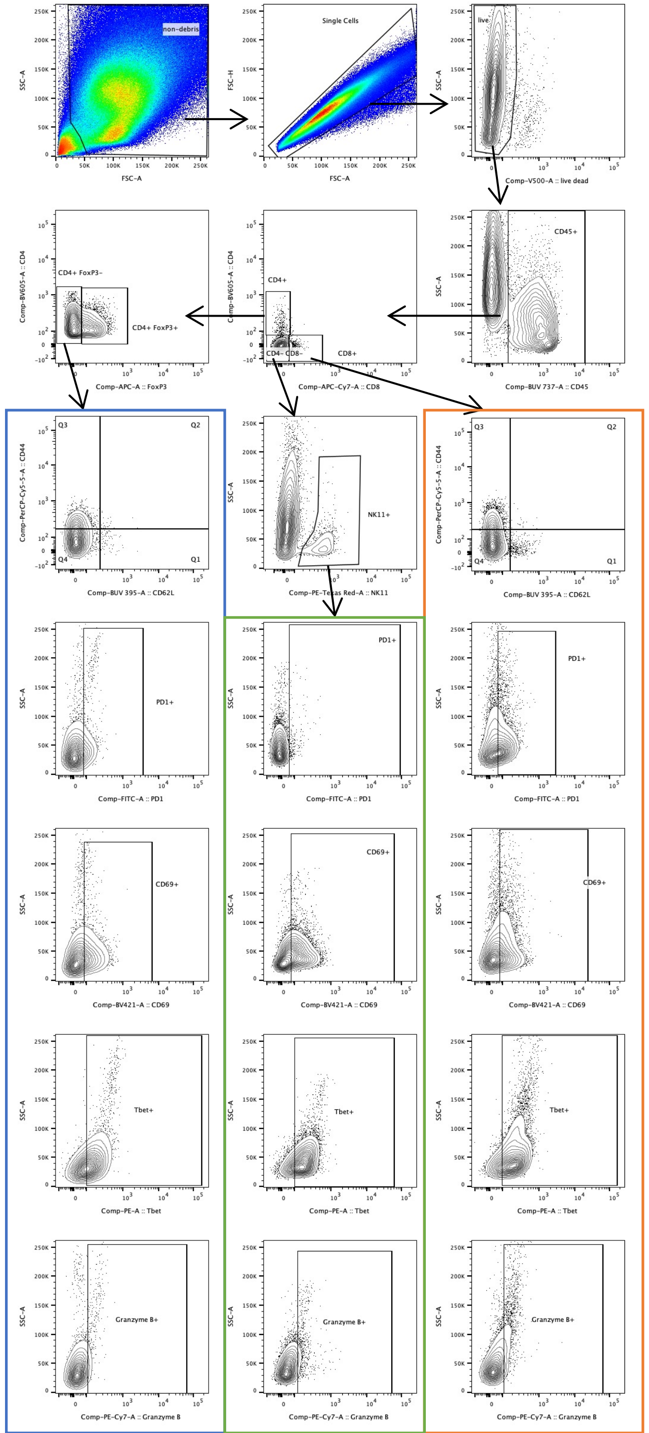

B

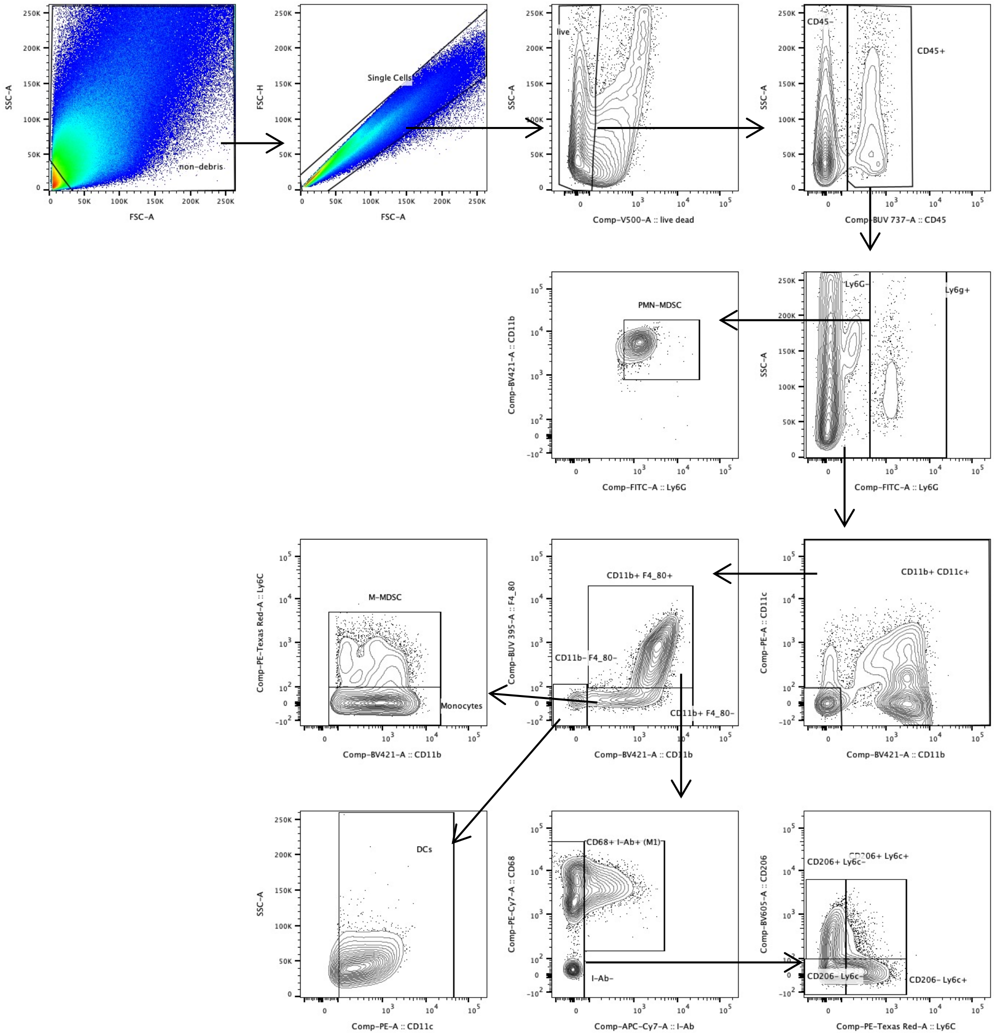

Figure S1. Gating schematics for flow cytometry analysis of the B16 and BPR TME. The gating strategy for T cells and NK cells is shown in (**A**), while the gating strategy for myeloid cells is shown in (**B**).

Fig. S2, Filderman et al.

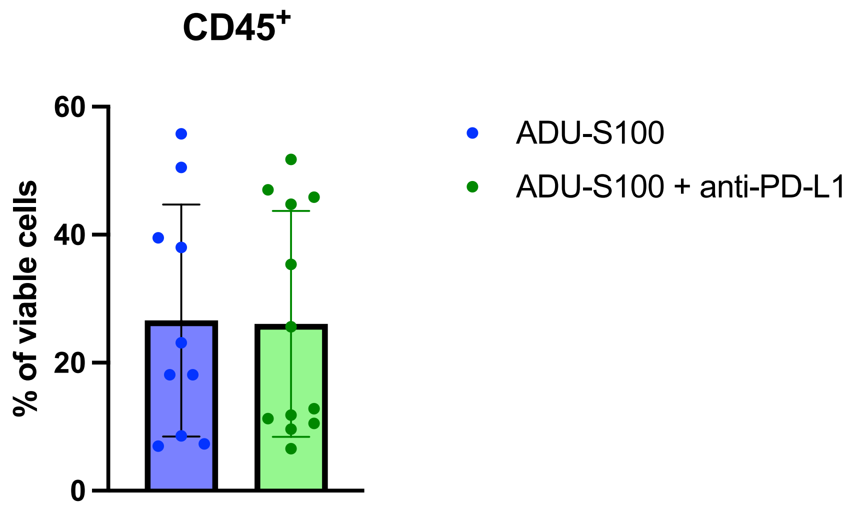

Figure S2. No significant changes were observed in CD45^{+} immune cell infiltrates in B16 melanomas treated with ADU-S100 + ISG inhibitors vs. ADU-S100 alone. Mice bearing established s.c. B16 melanomas (as described in **Fig. 2**) were treated with ADU-S100 (i.t.) + anti-PD-L1 antibody (i.p.) vs. ADU-S00 (i.t.) alone. On day 7 after the initiation of treatment, tumors were harvested, dissociated into single cell suspensions, and stained for CD45 expression and analyzed by flow cytometry. ns for combination treatment vs. ADU-S100 monotherapy.

Fig. S3, Filderman et al.

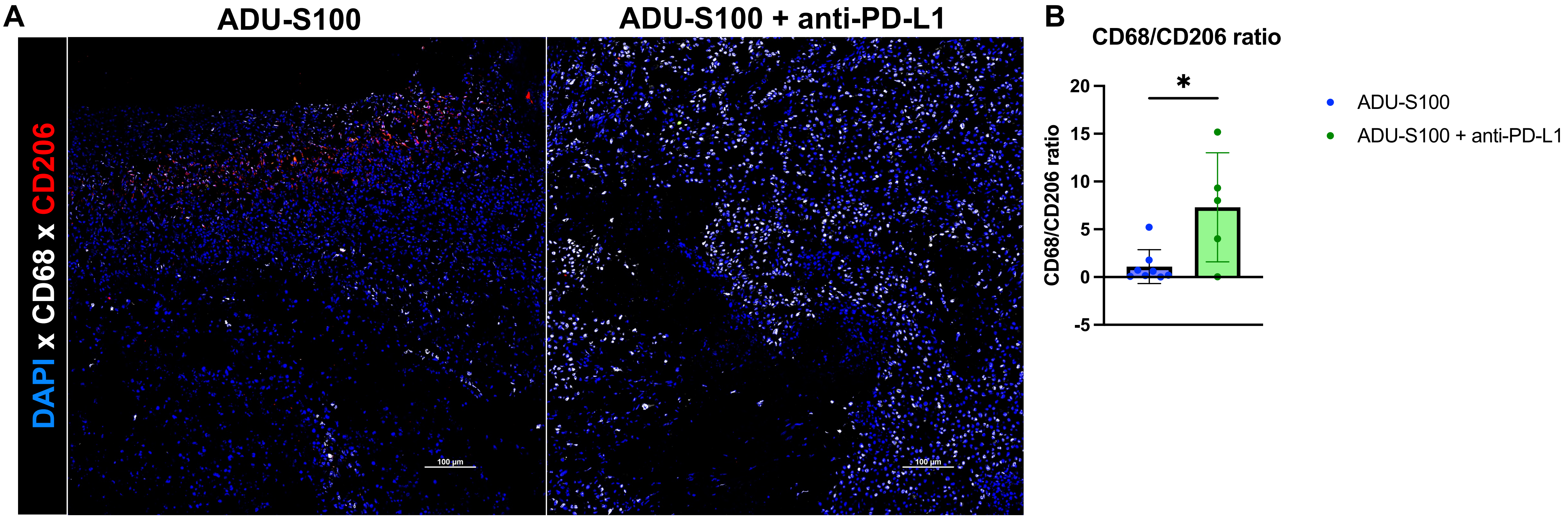

Figure S3. The TME of B16 melanoma-bearing mice treated with ADU-S100 + anti-PD-L1 contain more M1 macrophages vs. mice treated with ADU-S100 alone. Mice bearing established s.c. B16 melanomas (as described in **Fig. 2A**) were treated with ADU-S100 (i.t.) +/- anti-PD-L1 antibody (i.p.). On day 9 after the initiation of treatment, tumors were harvested, fixed, frozen, sectioned, and stained for markers of M1 (CD68) and M2 (CD206) macrophages. Representative images are shown in (A) with quantitation of the CD68/CD206 ratio for each treatment group shown in (B). (n=5, *p<0.05, unpaired two-tailed Student's t test)

Fig. S4, Filderman et al.

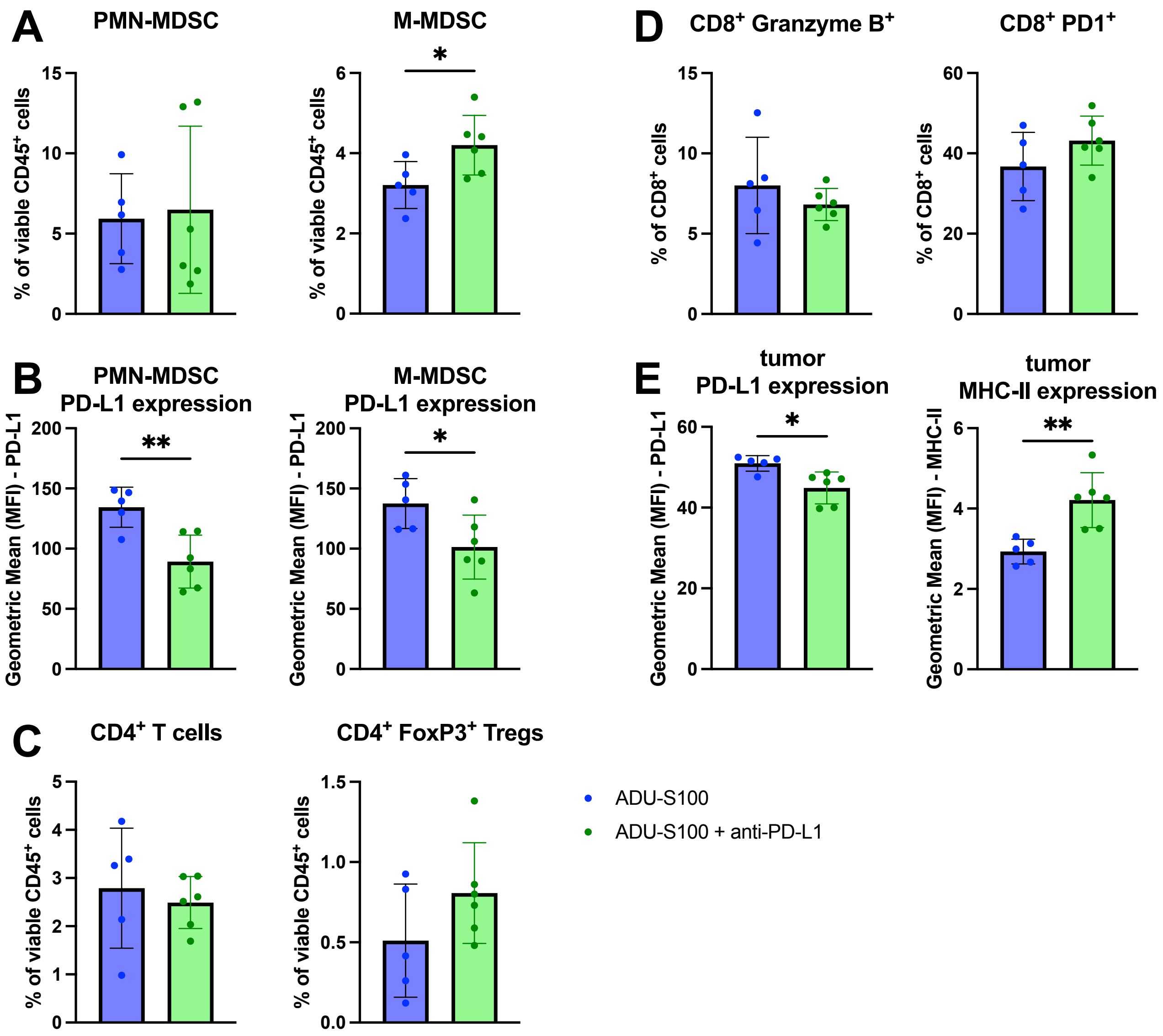

Figure S4. Treatment of established B16 melanomas with ADU-S100 + anti-PD-L1 antibody results in changes in immune cell status in the TME vs. treatment with ADU-S100 alone. Mice bearing established s.c. B16 melanomas (as described in **Fig. 2A**) were treated with ADU-S100 (i.t.) + anti-PD-L1 antibody (i.p.) vs. ADU-S00 (i.t.) alone. On day 7 after the initiation of treatment, tumors were harvested, dissociated into single cell suspensions, and stained using the indicated markers for flow cytometry analysis of (**A**) PMN-MDSC ($CD11b^+Ly6g^+$) and M-MDSC ($CD11b^+F4/80^-Ly6c^+Ly6g^-$), (**B**) PD-L1 expression amongst PMN-MDSC and M-MDSC, (**C**) $CD4^+$ T cells and $CD4^+Foxp3^+$ Treg cells, (**D**) $CD8^+$ T cells, and (**E**) PD-L1 and MHC-II expression amongst $CD45^{neg}$ tumor/stromal cells as outlined in Materials and Methods. (n = 6, *p<0.05, **p<0.01, unpaired two-tailed Student's t test).

Fig. S5, Filderman et al.

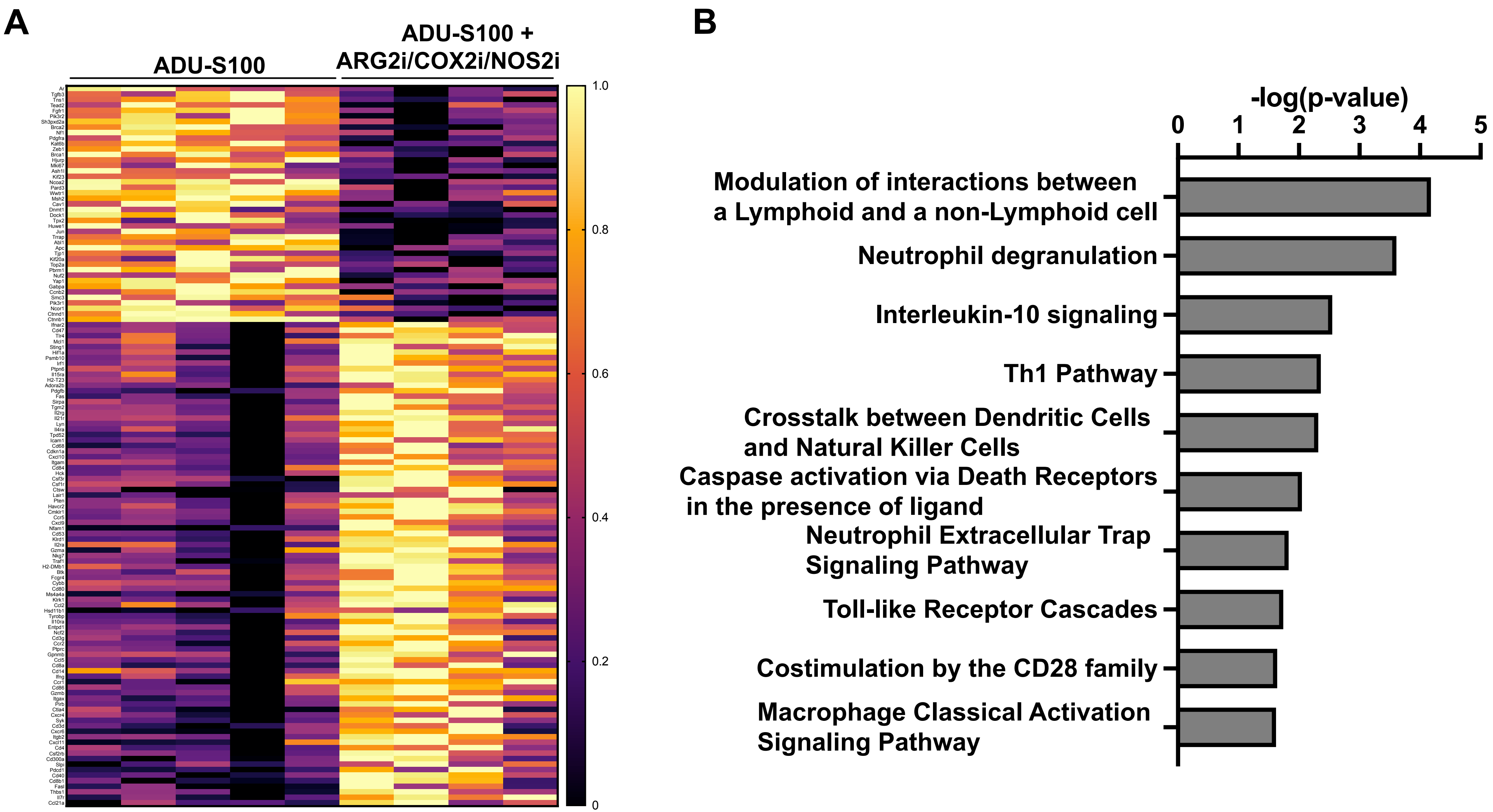

Figure S5. Treatment of BPR20 melanomas with ADU-S100 + ARG2i/COX2i/NOS2i leads to significant upregulation of genes associated with inflammation and functional anti-tumor immune responses. Mice with BPR20 melanomas were treated as described in **Fig 2**. On day 7 post-treatment initiation, tumors were harvested, dissociated into single cell suspensions, and RNA was isolated. RNA from the two treatment groups were analyzed using the Nanostring Tumor Signaling 360 panel, with genes significantly upregulated or downregulated reported in (**A**) and **Table S3**. A pathway analysis was then performed using the data from the Nanostring panel (**B**). All pathways listed have a z-score of >2.

Fig. S6, Filderman et al.

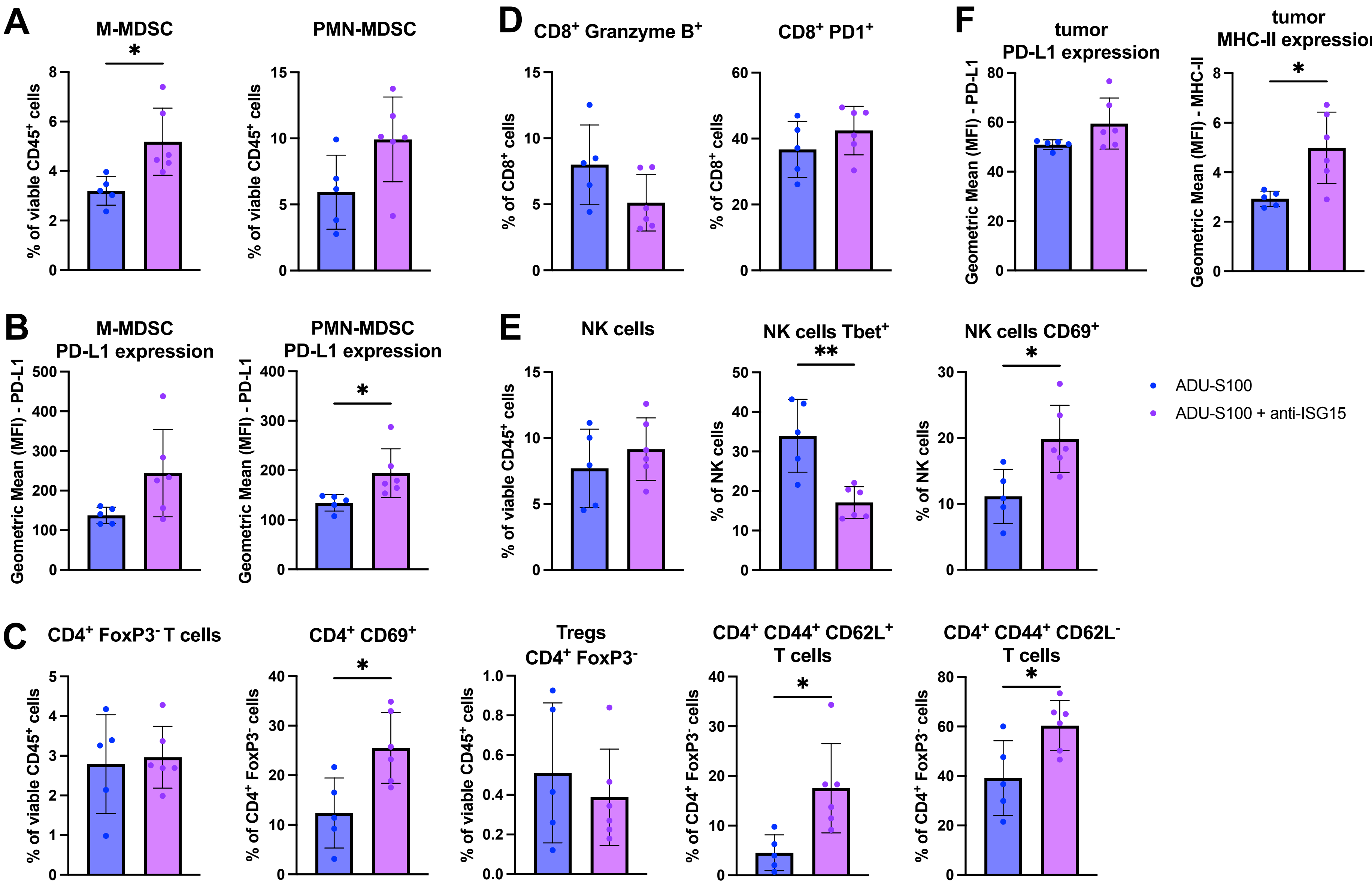

Figure S6. Treatment of established B16 melanomas with ADU-S100 + anti-ISG15 antibody results in changes in immune cell status in TME vs. treatment with ADU-S100 alone. Mice bearing established s.c. B16 melanoma (as described in **Fig. 4A**) were treated with ADU-S100 + anti-ISG15 antibody (both i.t.) vs. ADU-S00 (i.t.) alone. On day 7 after the initiation of treatment, tumors were harvested, dissociated into single cell suspensions, and stained using the indicated markers for flow cytometry analysis of (**A**) PMN-MDSC ($CD11b^+Ly6g^+$) and M-MDSC ($CD11b^+F4/80^-Ly6c^+Ly6g^-$), (**B**) PD-L1 expression amongst PMN-MDSC and M-MDSC, (**C**) $CD4^+$ T cells/subsets, (**D**) $CD8^+$ T cells, (**E**) NK cells/subsets, and (**F**) $CD45^{neg}$ tumor/stromal cells as outlined in Materials and Methods. (n = 6, *p<0.05, **p<0.01, unpaired two-tailed Student's t test).

Fig. S7, Filderman et al.

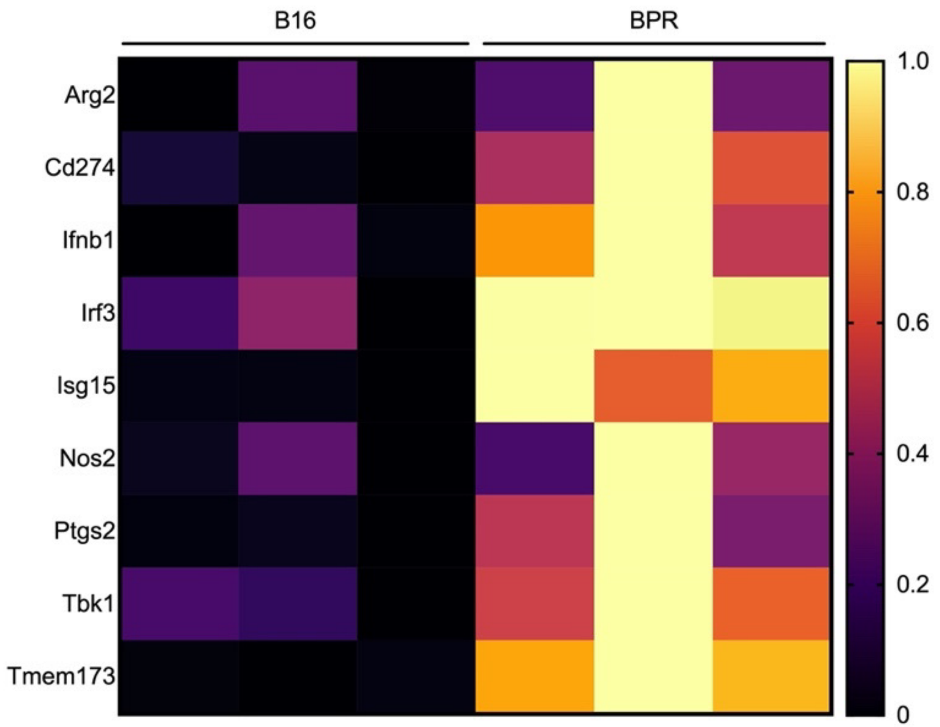

NO	YES	ADU-S100 +ARG2i/COX2i/NOS2i > ADU-S100
YES	NO	ADU-S100 + aPDL1 > ADU-S100
YES	NO	ADU-S100 + aISG15 > ADU-S100

Figure S7. No correlations were observed between baseline ISG and STING pathway component gene expression and enhanced response to combination STING agonist-based therapy in our two melanoma models. Mice bearing B16 or BPR melanomas were treated with two doses of PBS i.t. and tumors were collected 6 hours after the second dose. Gene expression levels of ISGs (Arg2, CD274, Isg15, Nos2, Ptgs2) and components of the STING pathway (Ifnb1, Irf3, Tbk1, Tmem173) were measured at baseline using the nCounter mouse PanCancer Immune Profiling Panel and compared between the two tumors.

Fig. S8, Filderman et al.

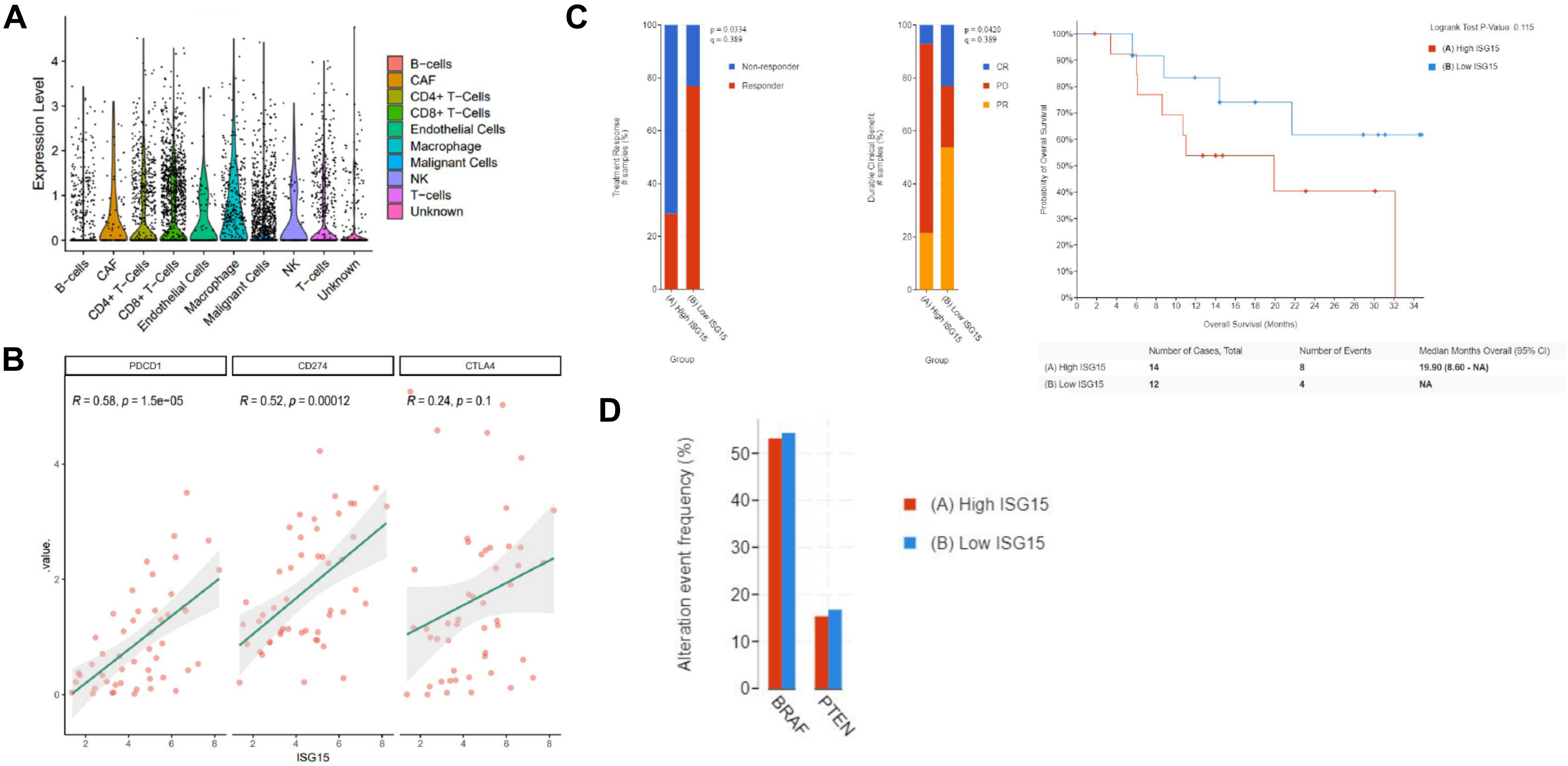

Figure S8. Profiling of *ISG15* in public-use melanoma data sets. In **A**, scRNAseq data (GSE115978) was used to profile *ISG15* expression in the indicated immune cell, stromal cell, and tumor cell populations in human melanoma specimens, with strongest expression noted for macrophages. In **B**, the GSE91061 melanoma data set was analyzed for correlative expression of *ISG15* with the indicated immune checkpoint molecules. In **C**, transcriptional and clinical profiling data from 27 melanoma patients treated with anti-PD1 (GSE78220) were analyzed for correlates with tumor *ISG15* expression. Low tumor *ISG15* expression trended for correlation with patient overall survival (p = 0.115), with patients with low tumor *ISG15* expression exhibited higher clinical response rates (p = 0.033) and ORR (p = 0.042). In **D**, the TCGA SKCM (n = 442) database failed to demonstrate melanoma variance in *ISG15* expression based on *BRAF* or *PTEN* mutation/loss status.
